# Fumigaclavine C from a Marine-Derived Fungus *Aspergillus Fumigatus* Induces Apoptosis in MCF-7 Breast Cancer Cells

**DOI:** 10.3390/md11125063

**Published:** 2013-12-13

**Authors:** Yong-Xin Li, S.W.A. Himaya, Pradeep Dewapriya, Chen Zhang, Se-Kwon Kim

**Affiliations:** 1 Marine Bioprocess Research Center, Pukyong National University, Busan 608-737, Korea; E-Mails: lyxycg@hotmail.com (Y.-X.L.); himayaswa@yahoo.com (S.W.A.H.); 2 Department of Chemistry, Pukyong National University, Busan 608-737, Korea; E-Mail: wgpradeep@gmail.com; 3 Shanghai Tenth People’s Hospital, Tongji University School of Medicine, Shanghai 200072, China; E-Mail: ahxczc@gmail.com

**Keywords:** fumigaclavine C, apoptosis, anti-proliferation, mitochondrial pathway, anti-cancer

## Abstract

Recently, much attention has been given to discovering natural compounds as potent anti-cancer candidates. In the present study, the anti-cancer effects of fumigaclavine C, isolated from a marine-derived fungus, *Aspergillus fumigatus*, was evaluated *in vitro*. In order to investigate the impact of fumigaclavine C on inhibition of proliferation and induction of apoptosis in breast cancer, MCF-7 cells were treated with various concentrations of fumigaclavine C, and fumigaclavine C showed significant cytotoxicity towards MCF-7 cells. Anti-proliferation was analyzed via cell mobility and mitogen-activated protein kinase (MAPK) signaling pathway. In addition, fumigaclavine C showed potent inhibition on the protein and gene level expressions of MMP-2, -9 in MCF-7 cells which were manifested in Western blot and reverse transcription polymerase chain reaction (RT-PCR) results. The apoptosis induction abilities of the fumigaclvine C was studied by analyzing the expression of apoptosis related proteins, cell cycle analysis, DNA fragmentation and molecular docking studies. It was found that fumigaclavine C fragmented the MCF-7 cell DNA and arrested the cell cycle by modulating the apoptotic protein expressions. Moreover, fumigaclavine C significantly down-regulated the NF-kappa-B cell survival pathway. Collectively, data suggest that fumigaclavine C has a potential to be developed as a therapeutic candidate for breast cancer.

## 1. Introduction

Breast cancer is one of the most common causes of cancer-related death in women. According to the World Health Organization, more than 1.2 million women are diagnosed with breast cancer each year worldwide [1,2]. Most of the present breast cancer chemopreventive and chemotherapeutic agents lead to undesirable side effects [3]. Therefore, the search for new agents derived from natural products with a fewer side effects should continue.

Marine fungi are a rich source of bioactive secondary metabolites including novel compounds that have unique structural features. Marine fungi have been widely studied for their bioactive metabolites, and these organisms have proved to be a rich, promising source of novel anticancer, antibacterial, antiplasmodial, anti-inflammatory and antitumor agents [4,5,6,7]. Therefore, bioactive compounds produced by marine fungi are of interest as new lead compounds in medicine.

*Aspergillus fumigatus* is a common environmental fungus and a significant cause of disease in immune-compromised patients and is responsible for up to 4% of deaths in tertiary hospitals in Europe [8]. Nevertheless, a number of bioactive compounds such as dioxopiperazine, alkaloids, dibenzofurans, and indole diketopiperazine have been isolated from *Aspergillus fumigatus* [9,10]. In this study, subsequent culturing and fractionation of the ethyl acetate (EtOAc) extract of *Aspergillus fumigatus* culture led to the isolation of fumigaclavine C as a major secondary metabolite. Fumigaclavine C is an indole alkaloid which was first isolated from the culture of *Cephalosporium* sp. IFB-018, an endophytic fungus from the rhizoma of a salinity-tolerant medicinal plant *Imperata cylindrica* by a column chromatography fraction of chloroform-methanol (1:1) extract [11,12]. Although this compound was discovered as early as 1977, its biological activity is seldom reported [13]. Its immunosuppressive activity against concanavalin A-induced hepatitis in mice by the mechanisms of inhibiting T cell proliferation, adhesion and TNF-α production has been reported previously, suggesting that fumigaclavine C may have a characteristic to inhibit the T-cell mediated immune response [14].

It is a well-known fact that alkaloids often possess significant physiological activities including anticancer and antitumor activity, and some of them are currently being used in clinical treatments. Moreover, in the broad range of alkaloids, indole-containing alkaloids have been reported as an interesting group of bioactive alkaloids and have frequently been isolated form marine organisms [15]. Ge and his research team [10] reported that two new alkaloids, which have a close similarity to fumigaclavine C in structure, showed selectively potent cytotoxicity against human leukemia cells (K562) with an IC_50_ value of 3.1 µM; however, detailed studies have not been reported yet. Therefore, in this study, we aimed to investigate the anti-cancer potential of fumigaclavine C while revealing the underlying molecular signaling pathways using a MCF-7 breast cancer cell model.

## 2. Results

### 2.1. Structural Elucidation of Fumigaclavine C

**Figure 1:**
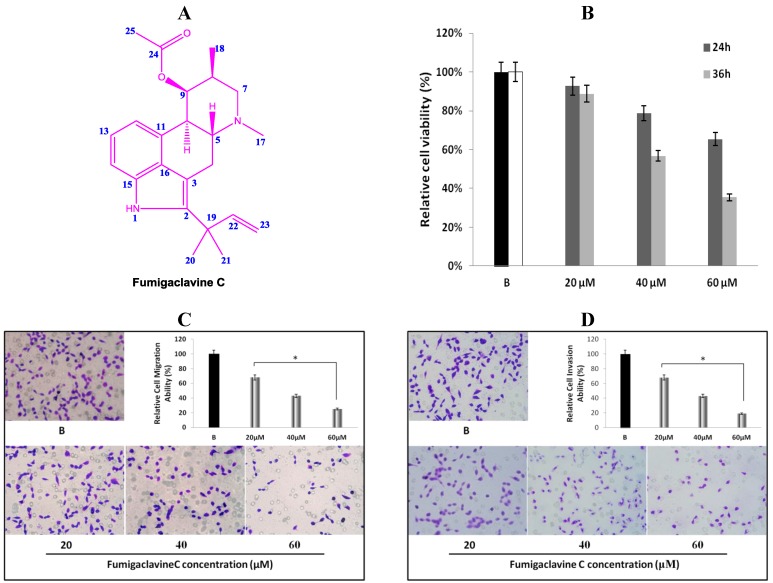
(**A**) Chemical structure of fumigaclavine C isolated from the marine-derived fungus *Aspergillus fumigatus*; (**B**) cytotoxic and anti-proliferation effect of fumigaclavine C on MCF-7 breast cancer cells. Briefly, MCF-7 cells were cultured in 96-well plates at a density of 5 × 10^3^ cells per well and treated with different concentrations (20 μM, 40 μM, and 60 μM) of fumigaclavine C for 24 h and 36 h; (**C**) and (**D**) effect of fumigaclavine C on MCF-7 cells migration and invasion. The results were observed with a microscope at 200× and the relatively blocked percentage (%) of migrated and invaded cells per field was assessed. Each value was expressed as the mean ± SD of triplicate experiments. * *p* < 0.05 as compared with blank groups.

The chemical structure of the isolated compound from broth extract of the marine-derived fungus was determined according to **1D**, **2D** nuclear magnetic resonance (NMR), and low-resolution electron ionization mass spectrometry (LREIMS) data, together with comparison with the data published previously [10]. The compound was identified as fumigaclavine C (15.8 mg), illustrated in Figure 1A.

Fumigaclavine C (15.8 mg): white powder; ^1^H-NMR (CD_3_OD, 400 MHz) δ 7.06 (1H, d, *J* = 8.0 Hz, H-12), 6.93 (1H, dt, H-13), 6.57 (1H, d, *J* = 7.1 Hz, H-14), 6.12 (1H, dd, *J* = 10.6 Hz, *J* = 6.5 Hz, H-22), 5.61 (1H, t, H-9), 5.03 (1H, dd, H-23), 5.06 (1H, t, H-23), 3.55 (1H, dd, H-10), 3.19 (1H, m, H-5), 2.71 (2H, m, H-7), 2.55 (2H, m, H-4), 2.41 (3H, brs, H-17), 2.07 (1H, m, H-8), 1.83 (3H, brs, H-25), 1.51 (6H, d, H-20, 21), 1.31 (3H, d, *J* = 7.5 Hz, H-18); ^13^C-NMR (CD_3_OD, 100 MHz) δ 172.6 (C-24), 147.6 (C-22), 138.5 (C-2), 134.5 (C-15), 129.3 (C-11), 129.0 (C-16), 122.5 (C-14), 112.8 (C-12), 111.4 (C-23), 109.1 (C-13), 106.1 (C-3), 72.8 (C-9), 63.3 (C-5), 58.9 (C-7), 43.9 (C-17), 40.5 (C-10), 40.3 (C-19), 34.5 (C-8), 28.9 (C-4), 28.1 (C-20), 28.0 (C-21), 20.9 (C-25), 16.9 (C-18). LREIMS *m/z*: 366.20 [M]^+^ (C_23_H_30_N_2_O_2_).

### 2.2. Anti-Proliferative Effect of Fumigaclavine C on MCF-7 Cells

The anti-proliferative effect of fumigaclavine C was tested on a cultured MCF-7 breast cancer cell line. Comparisons of the cell growth for 24 and 36 h with various concentrations of fumigaclavine C (20 μM, 40 μM, and 60 μM) are shown in Figure 1B. In a comparative analysis, fumigaclavine C showed significant high growth inhibitory effects on the MCF-7 cell line in a dose-dependent and time-dependent manner (*p* < 0.05). Fumigaclavine C inhibited the proliferation of MCF-7 cells with the viability percentages of 93%, and 89% (20 μM), 79%, and 57% (40 μM), and 65% and 35% (60 μM) at 24 h and 36 h, respectively, compared to the vehicle treated blank. Therefore, it was clear that fumigaclavine C had anti-proliferative effects on MCF-7 cells. Fumigaclavine C treatment (60 μM for 36 h) reduced the viable cell population up to 35% and thus fumigaclavine C treatment for 24 h was selected for further analysis.

### 2.3. Effects of Fumigaclavine C on Migration and Invasion of MCF-7 Cells

MCF-7 cells were treated with different concentrations (20 μM, 40 μM, and 60 μM) of fumigaclavine C for 24 h. It was observed that fumigaclavine C treatment reduced the cancer cell migration and invasion in a dose-dependent manner (Figure 1C,D). Interestingly, it was also observed that at the highest concentration (60 μM), fumigaclavine C almost completely blocked MCF-7 cell migration and invasion. Fumigaclavine C blocked the migration and invasion of MCF-7 cells with the blocking percentages of 38%, 29% (60 μM), and 25%, 19% (60 μM) at 24 h, respectively. The results indicate that fumigaclavine C has the ability to suppress MCF-7 cell migration and invasion.

### 2.4. Effect of Fumigaclavine C on MMP-2 and -9 Expressions in MCF-7 Breast Cancer Cells

**Figure 2:**
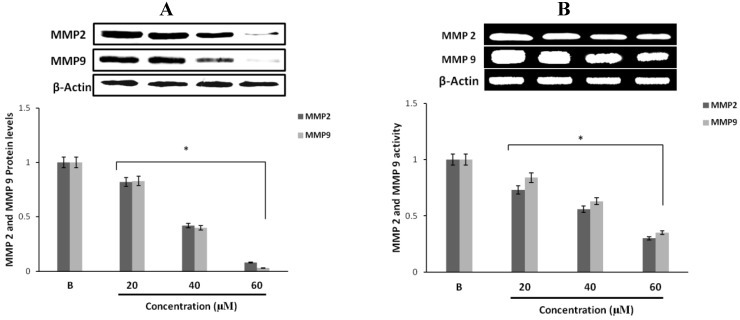
Inhibitory effect of fumigaclavine C on MMP-2 and -9 protein and mRNA expressions in MCF-7. (**A**) Protein expression level of MMP-2 and -9 in treated MCF-7 cells. Cell lysates were collected and subjected to sodium dodecyl sulfate polyacrylamide gel electrophoresis (SDS-PAGE) Western blot analysis using antibodies specific for MMP-2 and -9. Beta-actin was used as an internal control. (**B**) mRNA expression levels of MMP-2 and -9 in treated MCF-7 cells were analyzed by RT-PCR. Each value was expressed as the mean ± SD of triplicate experiments.* *p* < 0.05 as compared with blank groups.

Western blot results revealed that fumigaclavine C treatment of the MCF-7 cells resulted in an inhibition of protein expression of both MMP-2 and MMP-9. At the highest concentration (60 µM) of the compound there was a significant inhibition in both MMP-2 and MMP-9 and the inhibitory activity was significant (*p* < 0.05) from the concentrations of 20 to 60 μM (Figure 2A). These results suggest that fumigaclavine C effectively inhibits the MMP-2 and -9 activities and that this probably contributes to the anti-proliferative effects of fumigaclavine C.

To find out whether this inhibition of MMP-2 and -9 by fumigaclavine C are apparent at their gene levels, a reverse transcription polymerase chain reaction (RT-PCR) experiment was carried out using RNA isolated from MCF-7 cells treated with fumigaclavine C at different concentrations (20 μM, 40 μM, and 60 μM). Fumigaclavine C showed a clear inhibitory activity on the expression of both MMP-2 and -9 mRNAs in MCF-7 cells. The inhibition of MMP-2 and -9 gene expressions were observed in a concentration-dependent manner, where the inhibition was even lower than the blank group in MCF-7 cells at the highest concentration (60 μM) of the compound (Figure 2B). These results of the inhibition of MMP-2 and -9 mRNA expressions coincide with protein expression results suggesting that the MMP-2 and -9 were inhibited by the fumigaclavine C at both the protein and gene level. This clear inhibition of MMP-2 and -9 may be involved in the suppression of cell proliferation and migration.

### 2.5. Effect of Fumigaclavine C on ERK, JNK, and p38 MAPK Signaling Pathway

**Figure 3:**
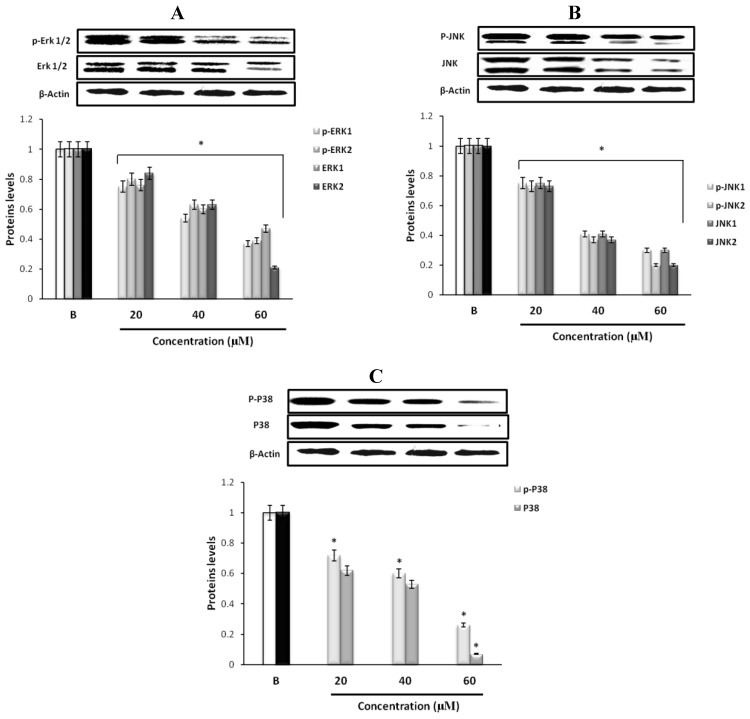
Effect of fumigaclavine C on expression of (**A**) p-ERK 1/2, ERK 1/2, (**B**) p-JNK, JNK, (**C**) p-p38, and p38 MAPK signaling pathways. Expression levels were assayed using Western blot following treatment with fumigaclavine C at different concentrations (20 μM, 40 μM, and 60 μM) for 24 h. Each value is expressed as the mean ± SD of triplicate experiments. *p < 0.05 as compared with blank groups.

To find out through which pathway fumigaclavine C blocked the expression of MMP, the effect of fumigaclavine C on the ERK 1/2, JNK, and p38 MAPK signaling pathways was analyzed. Treatment with fumigaclavine C at various concentrations (20 μM, 40 μM, and 60 μM) showed a dose-dependent inhibitory effect on the phosphorylation of ERK 1/2, JNK, p38 in MCF-7 cell line (Figure 3A–C).

These results demonstrated that the anti-proliferative effect of fumigaclavine C was mediated by blocking the signal transduction of MAPK pathway molecules, *i.e.*, ERK, JNK, and p38 MAPK signaling pathways in activated MCF-7 cells. Therefore it was clear that the possible molecular mechanism for the fumigaclavine C mediated inhibition of proteinases is probably the inhibition of the activation of these signaling cascades.

### 2.6. The Effects of Fumigaclavine C on the Cell Cycle of MCF-7 Cells

**Figure 4:**
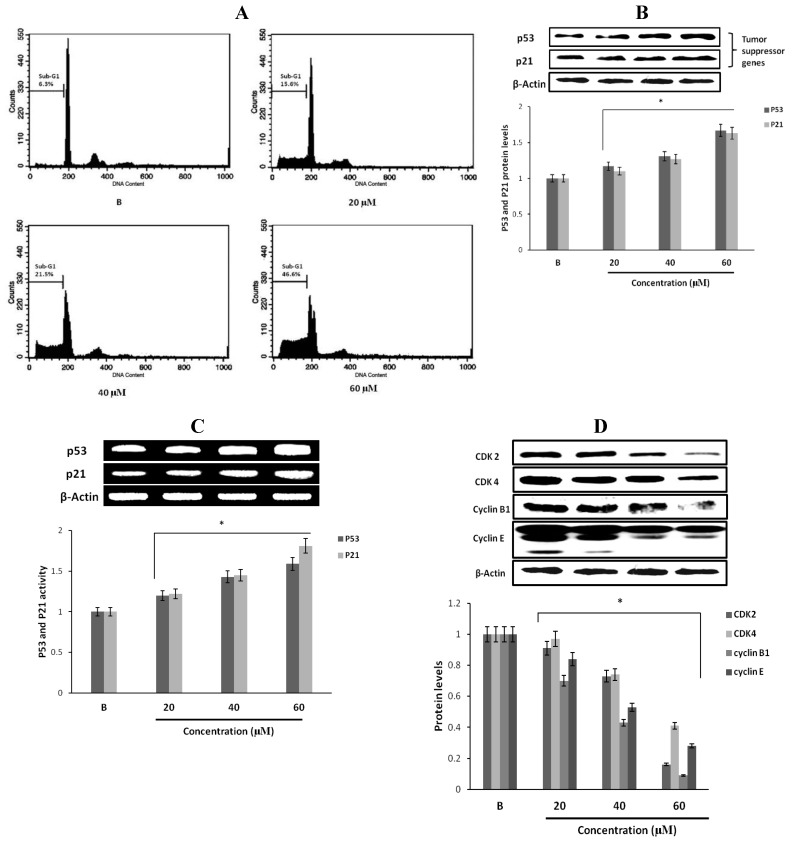
Effect of fumigaclavine C on cell cycle progression and cell cycle apparatus. (**A**) Cell cycle distribution pattern of fumigaclavine C treated (24 h) MCF-7 cells analyzed by fluorescence-activated cell sorting (FACS). Cells were fixed in 70% ethanol, re-suspended in PBS and stained with PI. After 24 h, a prominent sub-G1 peak could be seen in the histogram. (**B**) and (**C**) protein and gene expressions of p53, and p21 in MCF-7 cells treated with fumigaclavine C. MCF-7 cells were treated with various concentrations of fumigaclavine C for 24 h under serum-free conditions. (**D**) Protein expressions of CDK2, CDK4, cyclin B1, and cyclin E in fumigaclavine C treated MCF-7 cells. The antibody bindings were detected by enhanced chemiluminescence reagent using luminoimager. Each value was expressed as the mean ± SD of triplicate experiments. * *p* < 0.05 as compared with blank groups.

To further study the mechanism responsible for fumigaclavine C induced growth inhibition, the effect of fumigaclavine C on the cell cycle distributions was analyzed by flow cytometry. As shown in Figure 4A, treatment of the MCF-7 breast cancer cells with various concentrations of fumigaclavine C for 24 h resulted in a significant dose-dependent induction of sub-G1 cell population. It indicated that fumigaclavine C induced apoptosis in MCF-7 cells where the sub-G1 cell percentages were 15.6% (20 μM), 21.5% (40 μM), and 46.6%, (60 μM) at 24 h, respectively.

### 2.7. Effect of Fumigaclavine C on p53 Family Gene Expression in MCF-7 Breast Cancer Cells

The tumor suppressor gene p53 plays an essential role in various types of anti-proliferation and apoptosis. And the induction of p21 is mostly mediated through a p53-dependent pathway. Therefore, we determined whether the tumor suppressor factors, p53 and p21, were involved in the anti-proliferative effect of fumigaclavine C. Treatment with different concentrations of fumigaclavine C activated p53 and p21 proteins and gene levels in a dose-dependent manner (Figure 4B,C). These results imply that a part of the anti-proliferative activity of fumigaclavine C is related to apoptosis through up-regulation of p53 and p21 levels.

### 2.8. Effect of Fumigaclavine C on Cyclin B1, Cyclin E, CDK2, and CDK4 Expression in MCF-7 Breast Cancer Cells

To further explore the underlying mechanism by which fumigaclavine C mediates cell cycle arrest, we examined the regulatory effects of fumigaclavine C on the expression of CDK2, CDK4, cyclin B1 and cyclin E, which control the cell cycle progression. As shown in Figure 4D, CDK2, CDK4, cyclin B1, and cyclin E were down-regulated to varying degrees by fumigaclavine C treatment. These results demonstrated that fumigaclavine C induced cell cycle arrest by changing the CDK2, CDK4, cyclin B1 and cyclin E protein expression levels.

### 2.9. The Morphological Changes and DNA Damages of MCF-7 Cells Observed with Hoechst 33258 Staining

**Figure 5:**
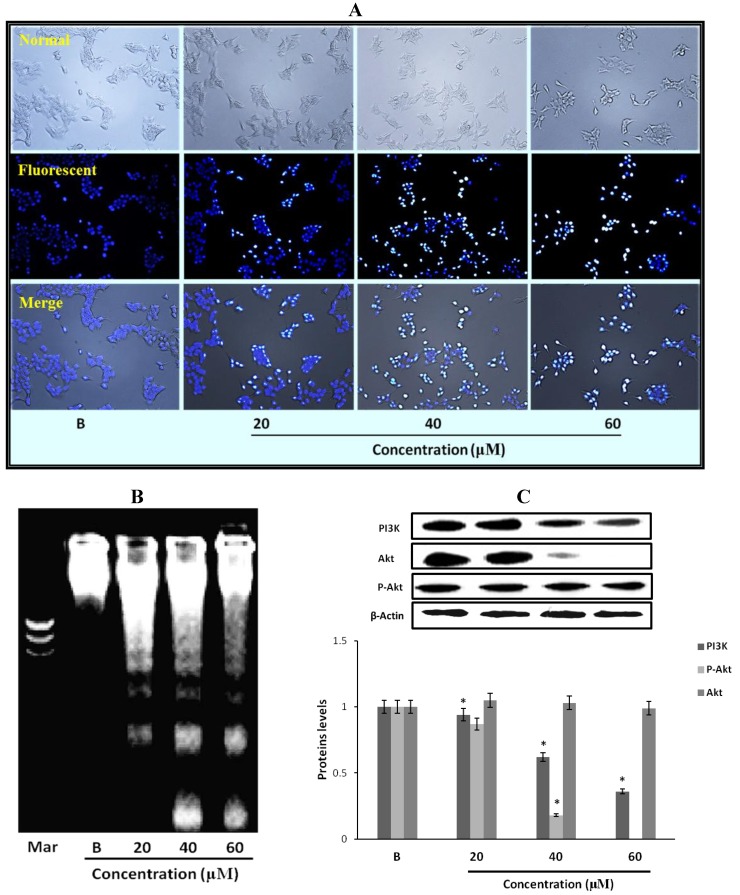
Effect of fumigaclavine C treatment on nuclear damage of MCF-7 cells. (**A**) Hoechst staining of MCF-7 cells treated with fumigaclavine C. For Hoechst staining, MCF-7 cells were cultured in 24-well plate and treated with fumigaclavine C for 24 h. (**B**) The effects of fumigaclavine C on the DNA fragmentation. (**C**) Western blot analysis of the expressions of PI3K, p-Akt, and Akt in MCF-7 cells treated with fumigaclavine C. MCF-7 cells were grown at 5 × 10^5^ cells/dish and treated with different concentrations of fumigaclavine C. Each value was expressed as the mean ± SD of triplicate experiments. * *p* < 0.05 as compared with blank groups.

To observe the morphological changes and DNA damage induced by the fumigaclavine C in MCF-7 cells, Hoechst 33258 staining was used as described earlier. The results are depicted in Figure 5A. MCF-7 cells were cultured in 24-well plate and treated with different concentrations (20 μM, 40 μM, and 60 μM) of fumigaclavine C for 24 h. The results were observed under fluorescence inverted microscope at 100× magnification. Clear morphological changes and DNA damage were observed with the treatment of the compound. Moreover, the DNA damage was dose dependently increased. The results indicate that fumigaclavine C potently damages DNA and thereby induces apoptosis in MCF-7 cells.

### 2.10. Fumigaclavine C Induced DNA Fragmentation in MCF-7 Cells

For further confirmation of the effect of fumigaclavine C on nuclear damage, DNA fragmentation of MCF-7 breast cancer cells was analyzed by an agarose gel electrophoresis. There was a dose dependant DNA fragmentation or laddering pattern in the cells treated with various concentrations of fumigaclavine C compared to the blank (Figure 5B). Hence, the results could imply that fumigaclavine C may have induced apoptosis and subsequent DNA damage.

### 2.11. Effect of Fumigaclavine C on PI3K/Akt Pathway in MCF-7 Breast Cancer Cells

In order to elucidate the specific intracellular signaling pathways involved in the growth inhibitory effects of fumigaclavine C in MCF-7 cells, the effect of fumigaclavine C on the expression of PI3K, p-Akt, and Akt, which can control cell apoptosis progression was investigated. As shown in Figure 5C, PI3K and Akt were down-regulated by the treatment of fumigaclavine C. These results demonstrate that fumigaclavine C induces cell apoptosis most probably via regulating the PI3K and p-Akt protein levels.

### 2.12. Effect of Fumigaclavine C on Bcl-2 Family Protein Expression in MCF-7 Breast Cancer Cells

**Figure 6:**
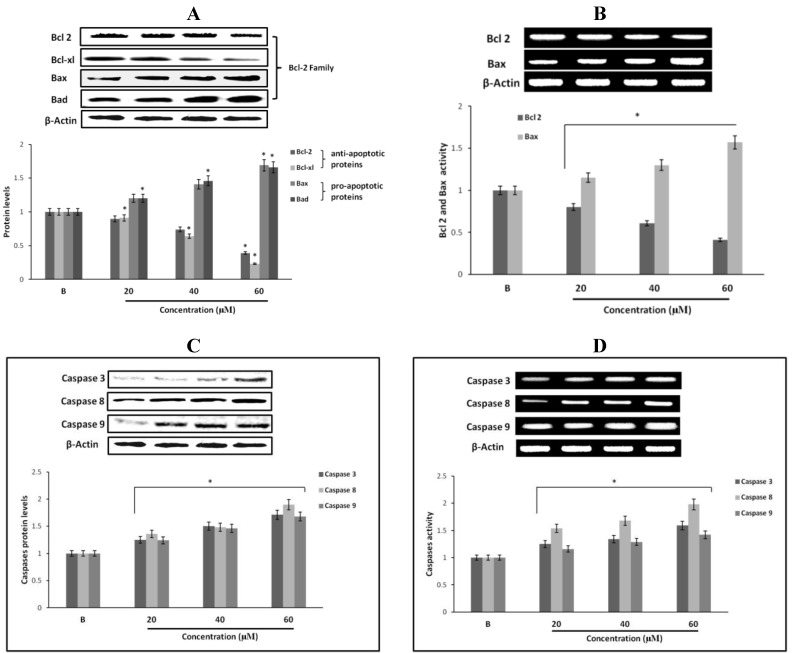
Effect of fumigaclavine C treatment on protein and gene expression in the apoptotic signaling cascade. (**A**) and (**B**) Protein and gene expressions of Bcl-2 family proteins in MCF-7 cells treated with fumigaclavine C. (**C**) and (**D**) effect of fumigaclavine C on the protein and gene expressions of caspase-3, -8, and -9 in MCF-7. Each value is expressed as the mean ± SD of triplicate experiments. *p < 0.05 as compared with blank groups.

Apoptotic genes regulate apoptosis by the activation of their pro- and anti-apoptotic products. Among them, Bcl-2 proteins are very important in apoptosis regulation. In order to confirm the expression levels of proteins and genes related to the induction of apoptosis, expression of Bcl-2, Bcl-xl, Bax and Bad in fumigaclavine C treated MCF-7 cells were analyzed using Western blot and RT-PCR analysis. As per the results fumigaclavine C exhibited the induction of apoptosis in MCF-7 cells by down-regulating the anti-apoptotic Bcl-2 and Bcl-xl levels as well as up-regulating the pro-apoptotic Bax and Bad levels at protein and gene levels (Figure 6A,B).

### 2.13. Effect of Fumigaclavine C on Caspase-3, -8, and -9 Expression in MCF-7 Breast Cancer Cells

To find out whether apoptosis is induced as an underlying mechanism of anti-proliferation of fumigaclavine C treatment, Western blot analysis was carried out to investigate the activation of caspase-3, -8 and -9. The results showed that the active caspase levels were increased by treatment with fumigaclavine C in a dose-dependent manner (Figure 6C). Furthermore, fumigaclavine C treatment more potently induced the expression of caspase-9 protein in MCF-7 cells compared to caspase-3 protein.

In order to analyze whether this activation of caspase-3, -8 and -9 and by fumigaclavine C occurs at their gene levels, a RT-PCR experiment was carried out using RNA collected from MCF-7 cells treated with fumigaclavine C at different concentrations (20 μM, 40 μM, and 60 μM). Fumigaclavine C showed a clear elevation in expression of caspase-9 and -3 mRNAs in MCF-7 cells. The activities of caspase-3, -8 and -9 gene expressions were observed in a concentration-dependent manner (Figure 6D). These expression levels of caspase-3, -8 and -9 mRNA coincide with protein expressions results, suggesting that the caspase-3, -8 and -9 were activated by the fumigaclavine C at both protein and gene levels. This clear activation of caspase-3, -8 and -9 may be involved in the induction of apoptosis.

### 2.14. Effect of Fumigaclavine C on Cytochrome C and Apaf-1 Protein Expression in MCF-7 Breast Cancer Cells

**Figure 7:**
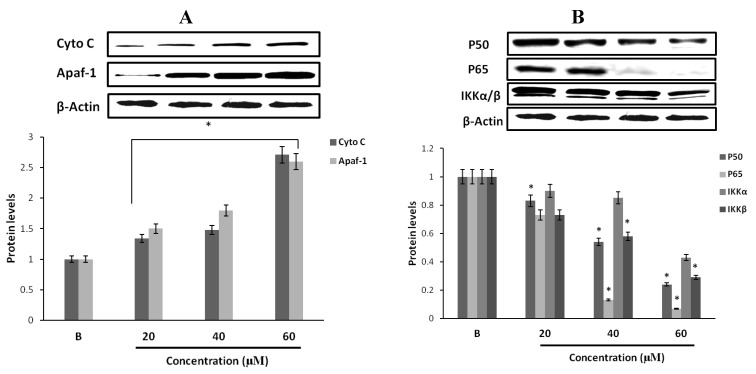
Protein expression levels of cell survival signaling molecules of MCF-7 cells. (**A**) Western blot analysis of the expression of cytochrome C and Apaf-1 in MCF-7 cells by fumigaclavine C. After incubation for 24 h, cell lysates were collected from tread and the same amount of proteins were subjected to Western blot using antibodies specific for cytochrome C and Apaf-1. β-actin was used as an internal standard. (**B**) Protein expressions of p50, p65, and IKK in MCF-7 cells by fumigaclavine C. Western blot analysis confirmed that the p50, p65, and IKK protein levels were down-regulated. MCF-7 cells were grown at 5 × 10^5^ cells/dish and treated with different concentrations of fumigaclavine C. The antibody bindings were detected by enhanced chemiluminescence reagent using luminoimager. Each value was expressed as the mean ± SD of triplicate experiments. * *p* < 0.05 as compared with blank groups.

In order to examine whether the activities of casapase-3 and caspase-9 were initiated due to mitochondrial release of cytochrome C and Apaf-1, the MCF-7 cells were exposed to fumigaclavine C in different concentrations (20 μM, 40 μM, and 60 μM). As shown in Figure 7A, we observed an increase of cytochrome C and Apaf-1 in the cytosolic fraction of fumigaclavine C treated MCF-7 cells. Mitochondrial release of cytochrome C and Apaf-1 resulted in the activation of caspase-9-mediated apoptosis.

### 2.15. Effect of Fumigaclavine C on p50, p65 and IKK Expression in MCF-7 Breast Cancer Cells

Transcription factor NF-κB (p50 and p65) is generally localized in the cytoplasm with its inhibitor IκB kinase (IKK). We investigated whether fumigaclavine C is associated with the inhibition of the activation of NF-κB and the NF-κB-dependent genes. The treatment with different concentrations (20 μM, 40 μM, and 60 μM) of fumigaclavine C inhibited the expression levels of p50 and p65 in a dose-dependent manner at transcriptional levels (Figure 7B). Down-regulation of IKK and up-stream activating kinases of NF-κB was also observed. Fumigaclavine C strongly suppressed NF-κB and IKK activation and this might have also been involved in the initiation of apoptosis.

### 2.16. Computational Modeling of the Fumigaclavine C Apoptosis Activity

**Figure 8:**
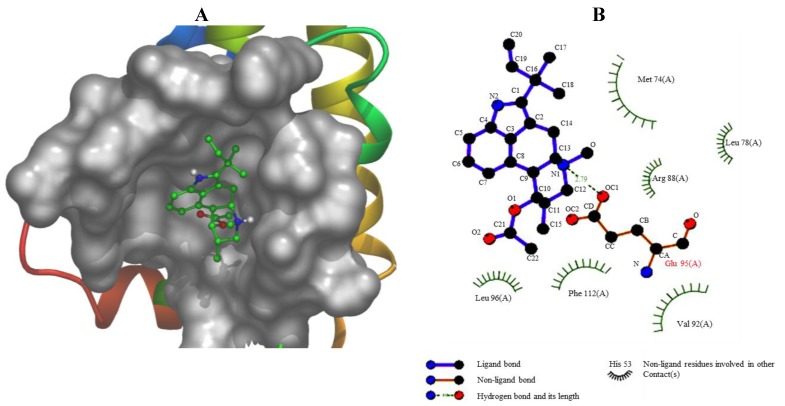
(**A**) The binding mode between Fumigaclavine C and 2w3l-Apoptosis. The interacting side chains of 2w3l-Apoptosis are displayed in surface mode. Fumigaclavine C is represented using balls and stick. The atoms of fumigalavine C are color-coded as follows: O, red; N, blue; C, green; H, white. (**B**) Schematic 2D plot showing intermolecular interactions.

The compound of fumigaclavine C was docked at the active site of the crystal structure of apoptosis regulator Bcl-2 (2w3L-Apoptosis). The computational docking study results are presented in Figure 8A,B. The schematic 2D plot shows intermolecular interactions of the representative fumigaclavine C with 2w3L-Apoptosis (Figure 8B). The nitrogen atoms of fumigaclavine C show an ionic interaction with GLU95 (2w3L-Apoptosis). The data show that fumigaclavine C has the lowest estimated (est.) energy of binding (−6.97) and est. inhibition constant (Ki = 7.75 µM) (Table 1), which is necessary for a strong interaction. In the binding mode (fumigaclavine C to 2w3L-Apoptosis), GLU95 residues in Bcl-2 were responsible for the hydrogen bonding and GLU95 alone was expected to make a polar interaction with fumigaclavine C. A hydrophobic interaction was the major interacting force brought up by MET74, LEU78, VAL92, LEU96, and PHE112 amino acid residues. The experimental results suggest that the fumigaclavine C can bind to the cleft of 2w3l-Apoptosis and interact with the key active-site residues GLU95, which can result in induced apoptosis activity.

**Table 1:** Docking results (fumigaclavine C was docked at the crystal structure of apoptosis regulator (2w3L-Apoptosis)).

Rank	Est. Free Energy of Binding	Est. Ingibition Constant, Ki	vdW + Hbond + desolv Energy	Electrostatic Energy	Total Internoiec. Energy	Frequency	Interact Surface
1	−6.97 kcal/mol	7.75 μM	−6.74 kcal/mol	−0.92 kcal/mol	−7.66 kcal/mol	40%	690.553
2	−6.60 kcal/mol	14.57 μM	−7.24 kcal/mol	−0.11 kcal/mol	−7.35 kcal/mol	10%	671.602
3	−6.56 kcal/mol	15.59 μM	−7.06 kcal/mol	−0.23 kcal/mol	−7.29 kcal/mol	10%	671.075
4	−6.34 kcal/mol	22.62 μM	−6.78 kcal/mol	−0.29 kcal/mol	−7.07 kcal/mol	30%	650.265
5	−5.85 kcal/mol	51.78 μM	−6.26 kcal/mol	−0.30 kcal/mol	−6.55 kcal/mol	10%	639.255

## 3. Discussions

Apoptosis is a programmed cell death mechanism recognized as a vital process in the regulation of tissue development and homeostasis, and a highly conserved mechanism throughout evolution [16,17]. Homeostasis between cell death and cell proliferation is required to maintain a normal state in all tissues [18,19]. The present study was designed to determine the anti-proliferative and apoptotic effects of fumigaclavine C isolated from a marine-derived fungus, *Aspergillus fumigatus*, on MCF-7 human breast cancer cells.

In recent years, interest in bioactive ingredients of marine fungi has been growing rapidly, as most of the fungal derived compounds are capable of inhibiting the growth and proliferation of cancer cells [20]. As shown in Figure 1B, fumigaclavine C showed significant cytotoxicity towards MCF-7 breast cancer cells and arrested progression of cell cycle at the G1 level inducing apoptosis in the cell. Even though similar cytotoxicity studies of effects of this compound on other cell lines have not been reported in the literature, Ge and colleagues [10] showed that 9-deacetylfumigaclavine C and 9-deacetoxyfumigaclavine C, which have a close similarity to fumigaclavine C in structure, have potent cytotoxicity towards human leukemia (K562), human nasopharynyeal epidermoid tumor (KB), and human breast adenocarcinoma (MCF-7) cells. Furthermore, previous studies have reported that fumigaclavine C acts against Con A-induced hepatitis in mice by inhibiting T cell proliferation, adhesion and TNF-α production [14]. Hence, it may be expected that fumigaclavine C might have more or less similar cytotoxic effects on other cancer cell lines. Moreover, Du and colleagues revealed that the compound does not exert any detectable cytotoxicity on RAW 264.7 cells at concentrations up to 100 μM [21]. These findings indicate that fumigaclavine C shows a selective toxicity towards cells, which is an interesting characteristics in chemo preventive agents, as most potent cytotoxic compounds result in several side effects due to lack of selectivity.

Here, we show that fumigaclavine C induces apoptosis in MCF-7 cells while inhibiting expression of relevant matrix metalloproteases (MMPs). Anti-apoptotic actions along with inhibition of MMP expression depend on the activation of the MAPK signaling pathway [22]. Several studies have shown that MMPs promote tumor proliferation and migration and therefore an anti-cancer compound with MMP inhibitory activity provides added advantage in chemoprevention. It has been found that metalloproteinase MMP-2 is responsible for increased invasiveness while MMP-9 expression in carcinoma cells offers survival advantage [23]. Our data clearly demonstrates that fumigaclavine C can inhibit MMP-2 and -9, which are known to be highly expressed in invasive cancer cells while suppressing the survival of cancer cell. A number of articles have shown that the MAPK signaling pathways play a considerable role in MMP expression and apoptosis related to cancer signaling cascades [24,25,26,27,28]. The results indicate that the inhibitory effect of fumigaclavine C on cell mobility, invasion and MMP-2, -9 expressions are due to the compound-mediated suppression of phosphorylation of the MAPK signaling molecules ERK, JNK and p38.

Tumor suppressor gene p53 plays an essential role in cell death and apoptosis [29]. When MAP kinases are activated, they function as effector protein kinases to phosphorylate a number of substrates including p53. The p53 is the up-stream molecule of cyclin dependent kinase inhibitor p21, and leads to many changes in cells such as cell growth differentiation and apoptosis [30,31]. Therefore, the p53 family was examined to determine apoptotic mechanisms in MCF-7 cells. Treatment with fumigaclavine C induced the up-regulation of cyclin dependent kinase inhibitor p21 as well as the down-regulation of cyclin B1, cyclin E, CDK2 and CDK4 levels.

Interestingly, recent findings have revealed that MAPK inhibitory compounds have a high potential to strengthen their apoptotic activity via regulating Bcl-2 family proteins, phosphatidylinositol PI3K/Akt and nuclear factor kappa B (NF-κB) [32,33]. Akt is an up-stream signaling intermediate molecule of NF-κB dependent cell survival gene expression, and activation of NF-κB and subsequent signaling molecules plays an important role in tumor cell growth, cell proliferation, tumor cell invasion and survival [34,35,36]. Inhibition of NF-κB activity has been identified as an apoptotic stimulus in a number of human cell lines [37]. NF-κB activation requires phosphorylation of IκBα by IκB kinases (IKKs). IκBα phosphorylation targets IκBα for ubiquitination and proteolytic degradation, releasing p50-p65 heterodimers to migrate into the nucleus and activate transcription [38,39]. In our results, it was clear that fumigaclavine C potently inhibited the activation of NF-қB and responsible gene expressions. A previous study reported a similar result, *i.e.*, that MMP-2 inhibitory apigenin isolated from *Anisomeles indica* showed a significant inhibition of NF-қB activation in MCF-7 cells [40].

Bcl-2 family proteins including Bcl-xl and pro-apoptotic members Bax and Bad are the most important regulators of apoptosis, and the activation of the signaling partly depends on the Akt-NF-қB activation. The results indicated that fumigaclavine C markedly reduced the expression of anti-apototic protein Bcl-2 and Bcl-xl while upregulating Bax and Bad expressions. In the literature, it could be found that many indole alkaloid induced apoptosis in cancer cells by down-regulating apoptotic Bcl-2 family proteins in a NF-қB-dependent manner [41,42]. In addition, many researchers have found that Bcl-2 protein is a promising small molecular anticancer target because active compounds that can bind to hydrophobic surface pockets in Bcl-2 protein can promote apoptosis. A computational docking study was performed to evaluate the affinity of fumigaclavine C to Bcl-2 protein. In the field of molecular modeling, docking is a method which predicts the preferred orientation of one molecule to a second when bound to each other to form a stable complex. Docking is frequently used to predict the binding orientation of small molecular drug candidates to their protein targets in order to predict the affinity and activity of the small molecule. Hence docking plays an important role in the rational design of drugs [43]. Interestingly, our molecular docking study showed that fumigaclavine C had a comparatively high capacity to bind with GLU95 (2w3L-Apoptosis) having the lowest est. energy of binding (−6.97) and est. inhibition constant (Ki = 7.75 µM). This might be the reason why fumigaclavine C showed a considerably high apoptotic effect on MCF-7 cells. Several findings support this argument showing that small molecules such as tetrocarcin A and antimycin A may have the ability to recognize surface pockets of Bcl-2 and Bcl-xl, respectively, and expedite apoptosis [44].

Activation of caspases is affected by Bcl-2 family proteins, which play a central role in the mitochondrial apoptosis pathway [45,46]. Apoptosis is induced via two main pathways involving either the mitochondria (the intrinsic pathway) or the activation of death receptors (the extrinsic pathway). Both pathways converge to induce the activation of caspases, the final executioners of cell death [47]. Caspases play essential roles in cells for apoptosis and have been termed "executioner" proteins for their roles in the cell [48]. The apoptosome is a multiprotein complex comprising cytochrome C, Apaf-1, and caspase-9 that functions to activate caspase-3, down-stream of the mitochondria apoptosis pathway in response to apoptotic signals. Binding of cytochrome C and dATP to Apaf-1 in the cytosol leads to the assembly of a heptameric complex in which each Apaf-1 subunit is bound non-covalently to a pro-caspase-9 subunit via its respective CARD domains [49]. Caspase-9 is activated very early in the apoptotic cascade by cytochrome C, which is released from the mitochondria in response to apoptotic stimuli [50]. Activated caspase-9 then initiates the proteolytic activity of other down-stream caspases such as caspase-3. The results of this study indicate that fumigaclavine C induces executor caspases-3, -8 and -9 leading to apoptosis. The western blot analysis may suggest that activation of apoptotic caspases is mainly due to induced expression of apoptotic Bax and Bid proteins, which leads to the mitochondrial cell death pathway. As showed in the data reducing the expression of Bcl-2 promotes the activity of Bax and Bak that mediate apoptosis by triggering destabilization of the mitochondrial membrane and consequently release the cytochrome c from the mitochondria to the cytosol. The release of cytochrome c is the leading factor for the higher expression of apoptotic caspases, which was manifested in our data after treatment with fumigaclavine C [45]. Furthermore, the result was confirmed by characteristic patterns of DNA fragmentation and cell cycle arrest.

## 4. Materials and Methods

### 4.1. Materials

*Aspergillus fumigatus* was isolated from the surface of marine green algae, collected at Seosaeng-myeon, Ulsan in the Republic of Korea in 2009. The fungal strain was cultured in YPG medium (1% yeast extract, 2% peptone, 10% glucose, 60% seawater, and 40% distilled water), and the fungal strain stored in 10% glycerol with the YPG medium at −78 °C for further experiments.

### 4.2. Chemicals

Extraction of the bioactive compound from *Aspergillus fumigatus* was performed using the extraction unit (Dongwon Scientific Co, Seoul, Korea). Silica gel 60 (230–400 mesh, Merck KGaA, Darmstadt, Germany), sephadex LH-20 (Sigma, St. Louis, MO, USA), YMC gel ODS-A 12 nm S-150 µm (YMC Co. Ltd., Kyoto, Japan) and thin-layer chromatography (TLC) plates (Kieselgel 60 F254, 0.25 mm, Merck KGaA, Darmstadt, Germany) were used for column chromatography and analytical TLC, respectively. The culture medium: Yeast extract (Lab M Limited, Lancashire, UK), peptone (Lab M Limited, Lancashire, UK), d-(+)-glucose (Yakuri, Sendai, Japan), agar powder (Lab M Limited, Lancashire, UK), glycerol (Sigma, 99%, St. Louis, MO, USA), penicillin G (Sigma, St. Louis, MO, USA). Organic solvent: *n*-hexane, EtOAc, CH_2_Cl_2_, MeOH, (Duksan Pure Chemical, Ansan-si, Korea, 99.5%) were used for the extraction. Coloring reagent used for visualization of TLC was Ce(SO_4_)_2_ (Sigma, St. Louis, MO, USA). ^1^H NMR (400 MHz) and ^13^C NMR (100 MHz) spectra were recorded on a JEOL JNM-ECP 400 NMR spectrometer (JEOL, Tokyo, Japan), using the CD_3_OD (3.34 ppm in ^1^H and 49.86 ppm in ^13^C NMR) solvent peak as an internal reference standard. Mass spectra were recorded on a JEOLJMS-700 spectrometer (JEOL).

### 4.3. Extraction and Isolation

The fungal strain was cultured in YPG medium (1% yeast extract, 2% peptone, 10% glucose, 4% agar, 60% seawater, and 40% distill water). Further culturing for extraction of metabolites was completed on YPG broth which was inoculated with 20 mL of fungal spore into 1 L long-neck flat bottom flask. The fungus was cultured (30 L) at 25 °C pH 7.6 in YPG medium for 30 days. The culture broth was extracted (2.3 g) with EtOAc (1:1.5 v/v, 1:1 v/v, broth–EtOAc) two times. The extract was fractionated by silica gel flash chromatography (*n*-hexane:EtOAc and CH_2_Cl_2_:MeOH) to generate five fractions. Final purification of each fraction was performed using ODS column chromatography (H_2_O:MeOH), followed by HPLC (YMC ODS-A, MeOH) and obtained fumigaclavine C (15.8 mg ) as a pure compound.

### 4.4. Cell Culture

Human breast cancer cells MCF-7 were obtained from American Type Culture Collection (ATCC). The cells were routinely grown in Dulbecco’s modified Eagle’s medium (Gibco, New York, NY, USA) supplemented with 10% FBS (Gibco, New York, NY, USA), 100 μg/mL penicillin, 100 μg/mL streptomycin, at 37 °C in humidified incubator under 5% CO_2_. For experiments, cells were passaged at least for 5 times and detached with trypsin-EDTA.

### 4.5. Cell Viability (MTT) Assay

Cytotoxicity levels and anti-proliferative effects of the isolated compound from *Aspergillus fumigatus* on MCF-7 cells were measured using MTT (3-(4,5-dimethyl-2-yl)-2,5-diphenyl-tetrazoliumbromide) assay as described by Carmichael *et al.* [51]. In the testing, MCF-7 cells were cultured in 96-well plates at a density of 5 × 10^3^ cells/well. After incubation for 24 h, cells were washed with fresh medium and were treated with different concentrations of fumigaclavine C. After 24 h or 36 h, 100 μL of MTT (0.5 mg/mL, final concentration) solution was added to each well and incubated for another 4 h at 37 °C under 5% CO_2_. The MTT solution was removed and DMSO (100 μL) was added to each well. The amount of formazan salt was determined by measuring the OD at 570 nm wavelength by GENios^®^ microplate reader (Tecan Austria GmbH, Grödig, Austria). The data were expressed as means of at least three independent experiments. Each value was expressed as the mean ± SD of triplicate experiments.

### 4.6. Cell Migration and Invasion Assay

The migration and invasion assay was performed using a 24-well transwell chamber with polyvinylpyrrolidone-free polycarbonate membranes (8 μm pore size) as previously reported [52]. For migration assay, 200 μL cells (2.5 × 10^5^/mL in serum-free medium) were placed in the upper compartment of the migration chamber and placed in 750 μL culture medium (with 10% FBS) in the lower chamber. The transwell was placed into the lower chamber to incubate at 37 °C in 5% CO_2_ for 12 h. Then it was incubated in a FBS-free medium containing 0.2% BSA in the presence or absence of various concentrations (20 μM, 40 μM, and 60 μM) of fumigaclavine C, dissolved in 10% DMSO for 36 h at 37 °C. In 5% CO_2_, the invasive cells attached to the lower surface of the inserted membrane and were fixed with 4% formaldehyde, methanol and stained with 0.5% crystal violet for 10 min [53]. After incubation, the filter inserts were removed from the wells, and the cells on the upper side of the filter were removed using cotton swabs. For the invasion assay, cells were measured in 24-well matrigel-coated (BD, New Jersey, NJ, USA) invasion chambers, other experiment steps were done with same with migration assay. Finally, the migrating and invading cells were observed with an optical microscope (Leica Microsystems Wetzlar GmbH, Wetzlar, Germany) at 100× magnification and the number of cells in four randomly selected microscopic bright fields per membrane was counted.

### 4.7. Hoechst 33258 Staining Assay

MCF-7 cells were cultured in 24-well plates at a density of 2 × 10^5^ cells/well. After the cells were treated with fumigaclavine C for 24 h, cells were fixed with 3.7% formaldehyde in PBS at room temperature for 20 min, the formaldehyde was aspirated and the cells were washed three times with PBS, cold (−20 °C) methanol (100%) was added and then left at room temperature for 20 min, then stained with Hoechst 33258 (0.12 μg/mL), and then subjected to fluorescence microscopy.

### 4.8. Cell Cycle Assay

Cell cycle analyses were performed on propidium iodide-stained nuclei by using CellQuest software on a FACSCalibur flow cytometer (Becton-Dickinson Biosciences, San Jose, CA, USA). MCF-7 cells were cultured in six-well plates at a density of 5 × 10^5^ cells/well. After the cells were treated with fumigaclavine C for 36 h, cells were harvested by trypsinizing, pelleted by centrifugation for 5 min at 1000 rpm, and 1 × 10^6^ cells were resuspended in 0.5 mL ice cold PBS. Cells were then fixed by drop-wise addition to 70% ice-cold ethanol and stored overnight at 4 °C. The cell pellet was then resuspended in 0.5 mL staining solution containing 50 µg/mL of RNase A (Sigma, St. Louis, MO, USA) in 0.1% Triton X-100 and 100 µg/mL of propidium iodide (Molecular Probes, Eugene, OR, USA). Data were analyzed by single histogram statistics as described [54].

### 4.9. DNA Laddering Assay

DNA was isolated according to the method described by Shinzawa *et al.* [55]. Briefly, MCF-7 cells (1 × 10^6^ cells/mL) were cultured in 10 cm2 culture disks for 24 h and treated with different concentrations (20 μM, 40 μM, and 60 μM) of fumigaclavine C. The amount of 1 × 10^6^ cells/mL was incubated with the tail lysis buffer (20 μg/mL of protease K, 1 mM of Tris pH 8.0, 100 mM of NaCl, 10 mM of EDTA, and 0.5% of SDS) for 24 h at 37 °C. Chromosomal DNA was obtained by phenol/chloroform/isoamyl alcohol (25:24:1) extraction and ethanol (100%) precipitation. The samples in TE solution (10 mM of Tris-HCl (pH 8.0) and 1 mM of EDTA) with 1 μg/mL of RNase A were incubated for 1 h at 37 °C. Ten micrograms of DNA from each sample were subjected to electrophoresis on 1.5% agarose gel for 2 h at 100 V and the gel stained with 1 mg/mL ethidium bromide and visualized by UV light using AlphaEase gel image analysis software (Alpha Innotech, Santa Clara, CA, USA).

### 4.10. Reverse Transcription-PCR (Polymerase Chain Reaction)

The total RNA from the MCF-7 cell line cultured in 10 cm dishes for 36 h was isolated using Trizol reagent (Invitrogen Co., Carlsbad, CA, USA) according to the supplier’s protocol and measured at 260 nm. In brief, 2 μg of total RNA was used to synthesize first-strand cDNAs with a kit (Promega, Madison, WI, USA), which synthesis cDNA with long mRNA templates (>5 kb) [56]. PCR was then carried out in 50 μL of reaction volumes containing RNA PCR buffer, 2.5 mM MgCl_2_, 0.2 μM of each primer, and 2.5 units of Taqpolymerase. Samples were pre-denatured at 94 °C for 4 min, followed by amplification at 94 °C for 1 min, at 55 °C for 30 s, and at 72 °C for 1 min for 30 cycles, followed by a final 10 min extension step at 72 °C. The primers for β-actin were forward primer 5′-AGC-CAT-GTA-CGT-AGC-CAT-CC-3′ and reverse primer 5′-TCC-CTC-TCA-GCT-GTG-GTG-GTG-AA-3′; the primers for MMP-2 were forward primer 5′-CAC-CTA-CAC-CAA-GAA-CTT-C-3′ and reverse primer 5′-AAC-ACA-GCC-TTC-TCC-TCC-TG-3′; the primers for MMP-9 were forward primer 5′-TTG-AGT-CCG-GCA-GAC-AAT-CC-3′ and reverse primer 5′-CCT-TAT-CCA-CGC-GAA-TGA-CG-3′; the primers for caspase-3 were forward primer 5′-GAA-CTG-GAC-TGT-GGC-ATT-GA-3′ and reverse primer 5′-TGT-CGG-CAT-ACT-GTT-TCA-GC-3′; the primers for caspase-8 were forward primer 5′-CAT-CCA-GTC-ACT-TTG-CCA-GA-3′ and reverse primer 5′-GCA-TCT-GTT- TCC-CCA-TGT-TT-3′; the primers for caspase-9 were forward primer 5′-TGG-ACG-ACA-TCT-TTG-AGC-AG-3′ and reverse primer 5′-GCA-AGA-TAA-GGC-AGG-GTG-AG-3′; the primers for Bax were forward primer 5′-TGC-CAG-CAA-ACT-GGT-GCT-CA-3′ and reverse primer 5′-GCA-CTC-CCG-CCA-CAA-AGA-TG-3′; the primers for Bcl-2 were forward primer 5′-CGC-ATC-AGG-AAG-GCT-AGA-GT-3′ and reverse primer 5′-AGC-TTC-CAG-ACA-TTC-GGA-GA-3′; the primers for p53 were forward primer 5′-GCG-CAC-AGA-GGA-AGA-GAA-TC-3′ and reverse primer 5′-CTC-TCG-GAA-CAT-CTC-GAA-GC-3′; the primers for p21 were forward primer 5′-CTG-TCA-CAG-GCG-GTT-ATG-AA-3′ and reverse primer 5′-TGT-GCT-CAC-TTC-AGG-GTC-AC-3′. Polymerase chain reaction products electrophoresed on 1.5% argarose gels were visualized by ethidium bromide staining and quantified using AlphaEase^®^ gel image-analysis software (Alpha Innotech, San Leandro, CA, USA).

### 4.11. Western Blot Analysis

Western blotting was performed according to standard procedures [57]. A total amount of 1 × 10^6^ of MCF-7 cells was treated with different concentrations (20 µM, 40 µM, and 60 µM) of the fumigaclavine C. The cells were lysed in RIPA lysis buffer containing 50 mM Tris-HCl (pH 7.5), 0.4% Nonidet P-40, 120 mM NaCl, 1.5 mM MgCl_2_, 2 mM phenylmethylsulfonyl fluoride, 80 µg/mL of leupeptin, 3 mM NaF and 1 mM DTT at 4 °C for 30 min. Cell lysates (about 20 μg of total proteins) were resolved on a 4%–20% SDS-page gel, electrotransferred onto a nitrocellulose membrane and blocked with 5% (w/v) non-fat dry milk in Tris-buffered saline and 0.1% Tween 20 for 1 h at room temperature. Different antibodies (Santa Cruz Biotechnology, Inc., Dallas, TX, USA) were used to detect the respective proteins using a chemiluminescent ECL assay kit (Amersham Pharmacia Biosciences, Buckinghamshire, UK), according to the manufacturer’s instructions. Blots were visualized using an LAS3000^®^ Luminescent image analyzer and protein expression was quantified by MULTI GAUGE V3.0 software (Fujifilm Life Science, Tokyo, Japan).

### 4.12. Docking Calculations

Docking calculations were carried out using DockingServer [58]. The MMFF94 force field was used for energy minimization of ligand molecule (Fumigaclavine C) using DockingServer [59]. Gasteiger partial charges were added to the ligand atoms. Non-polar hydrogen atoms were merged and rotatable bonds were defined.

Docking calculations were carried out on the crystal structure of apoptosis regulator Bcl-2 (2w3L-Apoptosis) protein model. Essential hydrogen atoms, Kollman united atom type charges, and solvation parameters were added with the aid of AutoDock tools. Affinity (grid) maps of 20 × 20 × 20 Å grid points and 0.375 Å spacing were generated using the Autogrid program [60]. AutoDock parameter set- and distance-dependent dielectric functions were used in the calculation of the van der Waals and the electrostatic terms, respectively.

Docking simulations were performed using the Lamarckian genetic algorithm (LGA) and the Solis & Wets local search method [61]. Initial position, orientation, and torsions of the ligand molecules were set randomly. Each docking experiment was derived from 10 different runs that were set to terminate after a maximum of 250,000 energy evaluations. The population size was set to 150. During the search, a translational step of 0.2 Å, and quaternion and torsion steps of five were applied.

## 5. Conclusions

**Figure 9:**
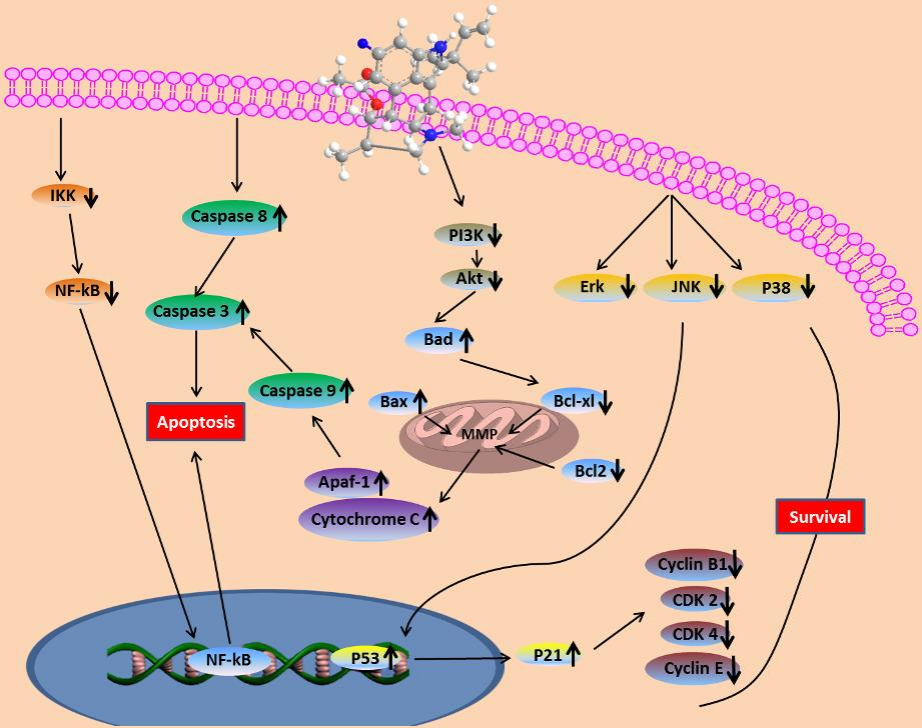
Fumigaclavine C-induced apoptosis in MCF-7 breast cancer cells through the mitochondrial pathway.

In summary, it was found in this study that fumigaclavine C isolated from a marine-derived fungus, *Aspergillus fumigatus*, shows potent anti-cancer activity. *In vitro* screening in MCF-7 human cancer cells showed that the compound induced apoptosis in the cells most probably via PI3/Akt and NF-κB signaling which lead to the activation of the mitochondrial cell death pathway. Moreover, the correlative mechanisms behind the anti-proliferation and apoptosis induction of fumigaclavine C were studied in detail (Figure 9). Collectively, it could be suggested that fumigaclavine C could be developed as potential therapeutic candidate in the treatment of breast cancer.
